# Ureteral metastasis as the first and sole manifestation of gastric cancer dissemination

**DOI:** 10.2478/v10019-010-0015-y

**Published:** 2010-04-23

**Authors:** Vesna Bisof, Antonio Juretic, Josip Pasini, Marijana Coric, Mislav Grgic, Marija Gamulin, Zoran Rakusic, Zdenko Krajina, Martina Basic-Koretic, Ana Misir, Ranka Štern-Padovan

**Affiliations:** 1 Department of Oncology, Clinical Hospital Centre Zagreb, Zagreb, Croatia; 2 Department of Urology, Clinical Hospital Centre Zagreb, Zagreb, Croatia; 3 Department of Pathology, Clinical Hospital Centre Zagreb, Zagreb, Croatia; 4 Clinical Institute for Diagnostic and Interventional Radiology, Clinical Hospital Centre Zagreb, Zagreb, Croatia

**Keywords:** ureteral metastasis, gastric cancer

## Abstract

**Background:**

Isolated ureteral metastasis from gastric cancer is extremely rare.

**Case report:**

We describe a 50 year old man with a history of subtotal gastrectomy who presented 4 years later with an ureteral metastasis. He was asymptomatic and diagnostic tests were performed due to the elevated creatinine level disclosed incidentally. The partial resection of distal right ureter as well as the resection of the right ureterovesical junction was performed with the implantation of double J stent. Histopathology revealed a metastasis of the adenocarcinoma that matched perfectly a tumour specimen from the gastric cancer surgery. It was first and isolated manifestation of gastric cancer dissemination.

**Conclusions:**

Although rare, the ureteral metastasis from gastric cancer can be the first, sole and asymptomatic manifestation of gastric cancer dissemination after a period of time.

## Introduction

The so-called true metastasis to the ureter from gastric cancer occurring through lymphatic and/or blood vessels is found to be very rare.^1^–^3^ There are also two other possibilities of the uretral obstruction: direct extension from the primary tumour, peritoneal deposit or lymph node metastasis of gastric cancer to the ureter – usually seen in the advanced cancer stage and autopsies^4^; and retroperitoneal fibrosis of the periureteral space induced by cancer cells.^5^

We report the case of a patient with ureteral metastasis as the first and sole manifestation of gastric cancer dissemination four years after he was first diagnosed with gastric cancer.

## Case report

A 50-year old man was admitted to the Department of Urology, Clinical Hospital Centre Zagreb in June 2008, due to the hydronephrosis and raised creatinine blood level disclosed incidentally during his rehabilitation from brain stroke from which he had suffered in May 2008.

**FIGURE 1.**
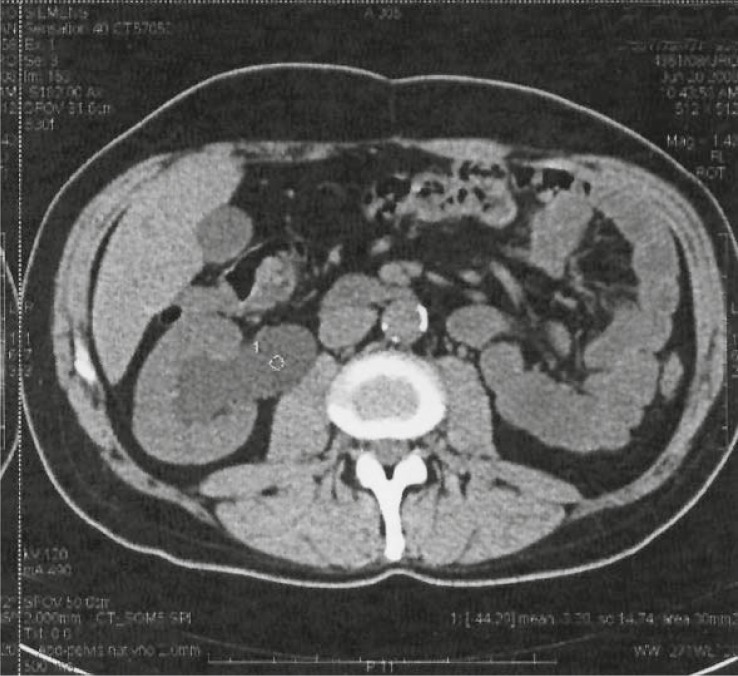
Multislice computed tomography (MSCT) - right hydronephrosis.

He had a history of subtotal gastrectomy for gastric cancer four years ago, stage T3N1M0. He received adjuvant chemo-radiotherapy. The patient had no pain at the admission. Routine blood test results were normal except elevated creatinine (192 μmol/L; normal range 63–107), urea (11.8 mmol/L; normal range 2.8–8.3) and C-reactive protein (CRP) (105 mg/L; normal range < 5) levels and mild anemia (hemoglobin 109 g/L; normal range 138–175). Urinalysis showed 3–7 erythrocytes and lot of leucocytes. Urine culture revealed Pseudomonas aeruginosa (10^3^ CFU/ml). Multislice computed tomography (MSCT) disclosed atrophic left kidney and right hydronephrosis (Figure 1). Cystoscopy indicated normal bladder. Right retrograde pyelography (RP) was not done successfully because of the obstruction found at the 3 cm from the right ureterovescial junction. Right antegrade pyelography showed hydrourether with contrast stop 4 cm below the right sacroilical joint.

On July 29, 2008, the partial resection of the distal right ureter as well as the resection of the right ureterovesical junction was performed with the implantation of a double J stent.

Histopathology revealed a metastasis of the adenocarcinoma. Fibromuscular and adipose tissue were infiltrated with tumorous tissue consisting of irregular glands lined with atypical colonic epithelial cells. No infiltration of a superficial transitional cell layer was found. The macroscopic observation of the periureteral region and of the retroperitoneal space did not reveal any pathology. Upon thorough comparison, tumour specimens of the resected ureter (year 2008) and gastric cancer (year 2004) were found to be completely identical (Figures 2a,b).

After the receipt of the histopathological report gastroscopy and colonoscopy were performed without any evidence of a tumour. Tumour markers were within the normal range: alpha- fetoprotein (AFP) (1.46 μg/L; normal range <13.4), carcinoembryonic antigen (CEA) (1.79 μg/L; normal range < 3.4), cancer antigen 19/9 (CA19-9) (16.93 kIU/L; normal range < 37), prostate-specific antigen (PSA) (1.44 μg/L, normal range < 4).

The patient was further transferred to the Department of Oncology for systemic chemotherapy. Unfortunately, he managed to receive only three cycles of chemotherapy (leucovorin, etoposide and fluorouracil) when presented with severe acute psychosis. Brain CT was without metastasis but his general and mental condition deteriorated and chemotherapy was never resumed. Finally, after several months his mental status gradually improved. Now, twelve months after the last chemotherapy he is well and without any signs of the disease.

## Discussion

Ureteral metastasis from distant organs is a rare event.^1^–^3^ The most common primary sites to metastasize to the ureter are breast, colon, prostate and cervix.^2^,^6^ MacKenzie and Ratner^1^ first proposed a criterion for the differentiation of a true metastasis from the direct extension of the tumour to the ureter. Later, Presman and Ehrlich^3^ modified the criterion as follows: “the demonstration of malignant cells in a portion of the ureteral wall together with the absence of any neoplasm in adjacent tissue”. Tumour in the ureteral wall without the invasion of the superficial transitional cell layer and the absence of any pathology in the periuretral and retroperitoneal space in our patient indicated the true uretral metastasis from gastric cancer.

Ureteral metastases from gastric cancer are extremely rare.^7^,^8^ Schlagintweit^9^ reported the first case of gastric cancer metastasizing to the ureter in 1911. Since then, cases have been occasionally reported. The majority of them were from Japanese population while reports from other populations were scarce.^10^ Shimoyama *et al.*^10^ reviewed 27 cases of the true ureteral metastasis from gastric cancer. The age of the patients ranged from 34 to 74 years with median age of 52 years. Eleven patients (41%) had previously undergone gastrectomy for gastric cancer. Our patient fitted the pattern.

Although rare on the whole, the ureteral metastasis from gastric cancer can be even the first manifestation of asymptomatic gastric cancer or the first and the sole manifestation of the gastric cancer dissemination after a period of time-as in our case.^10^–^12^ The prognosis is generally poor and the survival for more than 2 years has not been reported.^10^

There has been no report describing any effective therapy for this condition although there are some encouraging results with the multimodality treatment in another cases of patients with gastric cancer.^13^ The new regimens including docetaxel or oxaliplatin could show some benefit in the future. However, the pathohystology accomplished with immunohystochemistry, the establishing of extend of disease and the performance status still remain the main prognostic factors. They also enable the appropriate choice of the treatment.^14^

## Figures and Tables

**FIGURE 2A.**
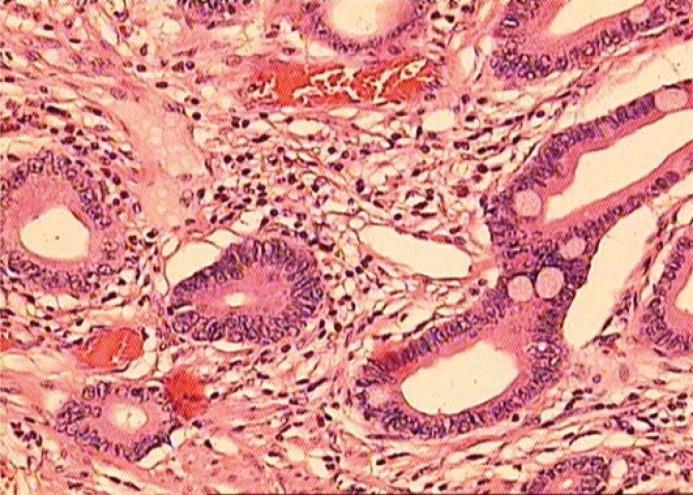
Microscopic appearance of stomach cancer. Hematoxylin and eosin staining (20 x magnification).

**FIGURE 2B.**
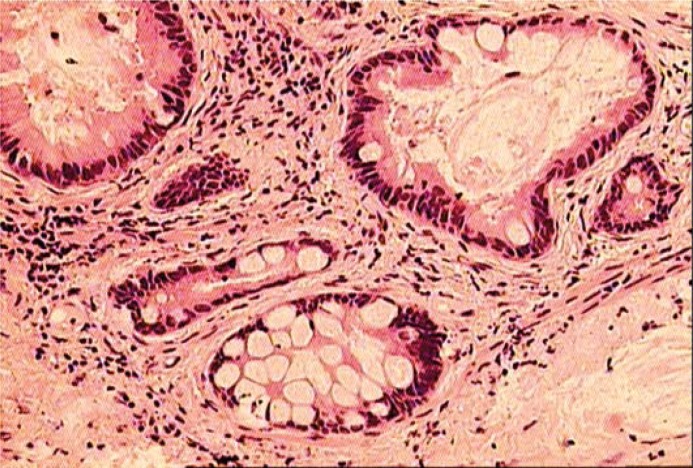
Microscopic appearance of the distal ureter cancer. Hematoxylin and eosin staining (20 x magnification).
